# Disrupting the Kinase–Phosphatase Equilibrium: A Unified Mechanism for Drug-Induced Eryptosis

**DOI:** 10.3390/hematolrep18050071

**Published:** 2026-09-29

**Authors:** Mohamed Jemaà, Yesmine Lahlaoui, Ghofrane Kitar, Khouloud Khalfaoui, Imen Moumni, Ines Safra

**Affiliations:** 1 Human Genetics Laboratory LR99ES10, Faculty of Medicine of Tunis, Tunis El Manar University, Tunis 2092, Tunisia; 2 Neurophysiology, Cellular Physiopathology and Valorisation of Biomolecules Laboratory LR18ES03, Faculty of Sciences of Tunis, Tunis El Manar University, Tunis 2092, Tunisia; 3 Department of Biology, Faculty of Sciences of Tunis, Tunis El Manar University, Tunis 2092, Tunisia; 4 Laboratory of Molecular and Cellular Hematology LR21IPT07, Pasteur Institute of Tunis, Tunis 1002, Tunisia

**Keywords:** drug-induced anemia, tyrosine kinase inhibitors, PI3K/AKT/mTOR, p38 MAPK, casein kinase 1α

## Abstract

The advent of targeted cancer therapies has revolutionized oncology, yet the off-target effects of these agents on healthy cells remain a significant clinical challenge. Anemia is one of the most frequent and debilitating adverse effects associated with pharmacological kinase inhibitors, often attributed to bone marrow suppression. However, accumulating evidence over the past fifteen years has illuminated an alternative mechanism: the direct induction of eryptosis, the programmed suicidal death of erythrocytes. This review provides a comprehensive analysis of the current state of knowledge regarding the induction of eryptosis by pharmacological modulators of the phosphorylation balance. We first detail the molecular machinery of eryptosis, then systematically evaluate the diverse classes of kinase inhibitors, from first-generation tyrosine kinase inhibitors to novel selective agents, as well as the emerging class of phosphatase inhibitors, examining the evidence for their eryptotic potential and the proposed molecular mechanisms driving this effect. Importantly, the majority of the evidence discussed derives from in vitro studies using erythrocytes from healthy donors, and we explicitly distinguish between experimentally demonstrated eryptosis and its proposed contribution to clinical anemia. Finally, we synthesize the clinical implications, discussing the impact on patient management, the potential for using eryptotic biomarkers as predictive tools for drug-induced anemia, and the broader pathophysiological consequences of chronic, drug-accelerated erythrocyte clearance. By integrating current mechanistic insights with clinical observations, this review underscores the importance of considering the kinase–phosphatase equilibrium as a potential determinant of drug safety and erythrocyte survival, and highlights the need for clinical studies to quantify the contribution of eryptosis to drug-induced anemia.

## 1. Introduction

The human body orchestrates a remarkable equilibrium between the production and clearance of its cellular constituents, a balance that is critical for homeostasis. Nowhere is this equilibrium more finely tuned than in the erythron, the population of red blood cells (RBCs). As anucleate, terminally differentiated cells, erythrocytes are uniquely vulnerable. Lacking the machinery for de novo protein synthesis and repair, they navigate a perilous 120-day lifespan through the vasculature, enduring cycles of osmotic stress, mechanical shear forces, and oxidative damage [1]. Their demise is not left to chance but is governed by a highly regulated, programmed form of cell death, a process termed eryptosis [2]. This evolutionarily conserved mechanism ensures the silent and controlled removal of senescent, damaged, or infected erythrocytes, thereby preventing the release of noxious intracellular contents (such as hemoglobin and heme) that would otherwise trigger inflammation, oxidative stress, and vascular dysfunction [3]. In healthy physiology, eryptosis is the quiet and efficient mechanism that clears the old to make way for the new, matching the rate of RBC production to clearance.

However, the same delicate molecular machinery that safeguards circulatory health can be hijacked or hyperactivated by a wide array of pathological conditions and xenobiotic compounds. The hallmarks of eryptosis, while ostensibly simple, represent the culmination of a complex signaling cascade that ultimately dismantles the erythrocyte’s structural and functional integrity. The central and initiating event is an increase in the cytosolic concentration of calcium ions (Ca^2+^) [4,5]. The erythrocyte maintains a steep Ca^2+^ gradient across its plasma membrane, with resting levels in the nanomolar range. Upon exposure to stressors, be it osmotic shock, energy depletion, oxidative stress, or, as is the focus of this review, pharmacological agents, the cell activates non-selective cation channels, most notably the transient receptor potential channel TRPC6 (and others), leading to a sustained Ca^2+^ influx [6].

This elevation in cytosolic Ca^2+^ acts as a master switch, setting in motion the two most distinctive morphological and biochemical hallmarks of eryptosis. Firstly, it activates the Ca^2+^-sensitive, intermediate-conductance potassium channel (KCa3.1, also known as Gardos channel) [7]. The efflux of K^+^ ions, accompanied by the obligatory loss of water and chloride, results in a profound cell shrinkage, a defining feature that distinguishes eryptosis from necrotic swelling [8]. Secondly, elevated Ca^2+^ leads to the breakdown of the asymmetric distribution of phospholipids in the plasma membrane. Under normal conditions, phosphatidylserine (PS), an “eat-me” signal, is actively sequestered in the inner leaflet of the lipid bilayer by the energy-dependent enzyme flippase (ATP11C) [9]. During eryptosis, Ca^2+^ inhibits the flippase and activates scramblases, a class of lipid-translocating enzymes. This results in the rapid, non-specific redistribution of phospholipids, leading to PS exposure on the outer membrane leaflet [10,11]. These exposed PS residues are recognized by specific receptors (e.g., on macrophages) that orchestrate the phagocytic clearance of the dying erythrocyte [12].

**Figure 1:**
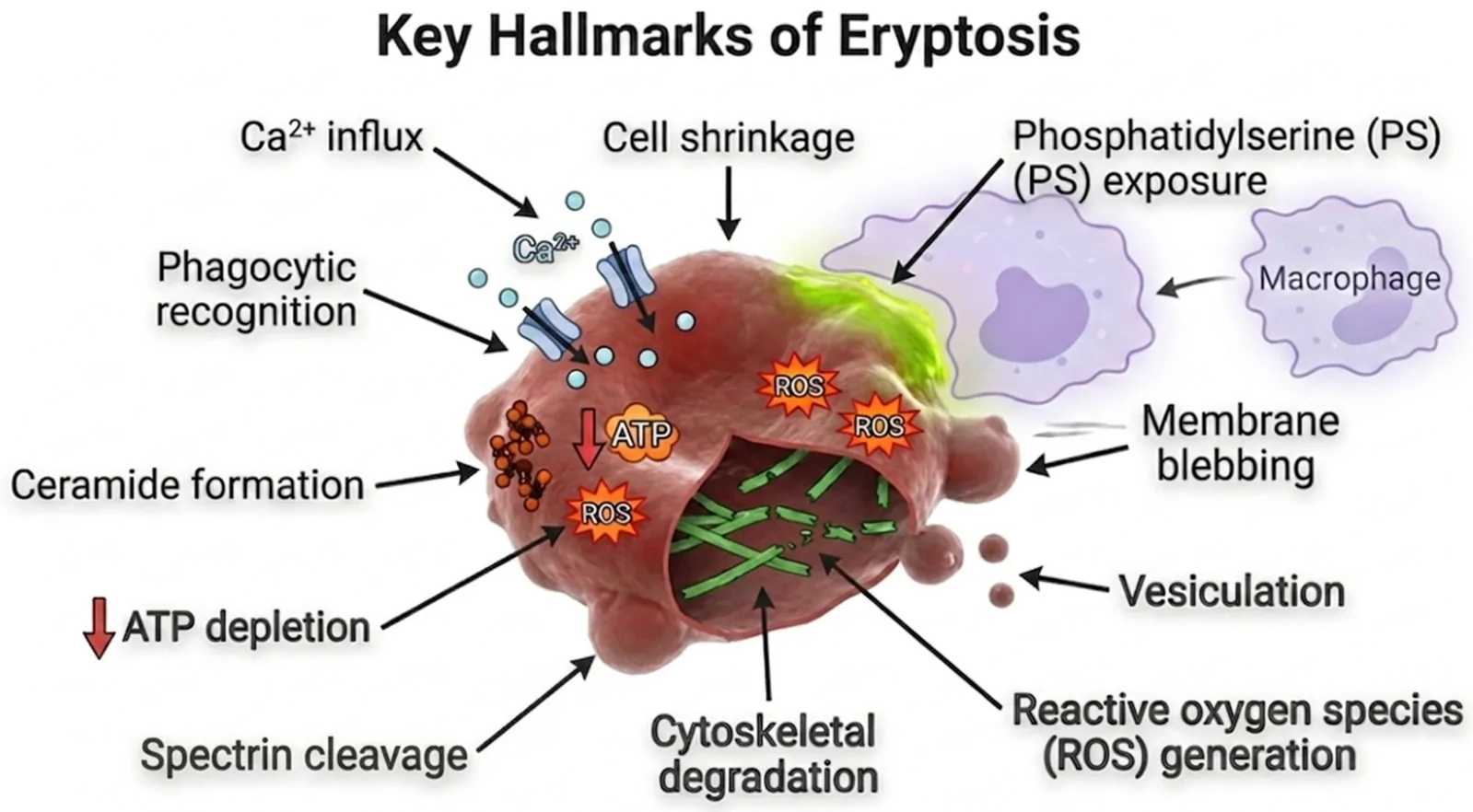
Key Hallmarks of Eryptosis.

Beyond these core features, eryptosis is characterized by additional hallmarks that contribute to its execution and clearance. These include vesiculation, the budding of microvesicles from the cell membrane that can shed damaged proteins or pathogens [13]; protease activation, involving calcium-dependent calpains that cleave cytoskeletal proteins like spectrin, contributing to membrane instability [14]; and the generation of reactive oxygen species (ROS), which often act as both triggers and amplifiers of the eryptotic cascade, establishing a positive feedback loop that can overwhelm the erythrocyte’s antioxidant defenses [15,16]. Furthermore, ceramide, a pro-apoptotic sphingolipid, accumulates in the erythrocyte membrane during eryptosis. Ceramide is generated primarily via the activity of acid sphingomyelinase, which can be stimulated by oxidative stress and Ca^2+^, and it contributes to membrane scrambling, PS exposure, and the formation of large, non-selective membrane pores, thereby reinforcing the eryptotic program [17,18] (Figure 1).

In recent decades, the pharmacological landscape has been revolutionized by the development and clinical deployment of small molecule kinase inhibitors. These compounds, designed to intercept aberrant signaling in malignancies and inflammatory disorders, have become cornerstones of modern precision medicine [17,18]. Drugs such as imatinib, dasatinib, sunitinib, sorafenib, and the ever-expanding generation of targeted agents have dramatically improved outcomes for patients with cancers like chronic myeloid leukemia, renal cell carcinoma, and non-small cell lung cancer [19]. Their mechanism of action is predicated on the selective inhibition of specific tyrosine or serine/threonine kinases, proteins that orchestrate cell proliferation, survival, and metabolism [20]. While their on-target efficacy is well-documented, the off-target effects of these agents on non-cancerous cells, particularly those of the hematopoietic system, are a growing area of concern and investigation.

Anemia is one of the most frequent and debilitating adverse effects associated with many tyrosine kinase inhibitors (TKIs) [21,22]. The etiology of this anemia is often attributed to bone marrow suppression, an on-target effect of inhibiting kinases crucial for erythropoiesis [23]. However, a growing body of evidence over the past fifteen years has illuminated an alternative, and potentially clinically significant, mechanism: the direct induction of eryptosis. While the quantitative contribution of this mechanism to clinical anemia remains to be established, the in vitro evidence suggests that eryptosis may exacerbate anemia independently of bone marrow suppression. Seminal studies by Föller et al. [24] and subsequent investigations demonstrated that dasatinib accelerate eryptosis, suggesting that this phenomenon is independent of their primary BCR-ABL target [25]. Many kinase inhibitors, irrespective of their primary molecular target, have since been shown to accelerate the premature death of circulating erythrocytes [26]. This phenomenon raises a critical paradox: while we are using these drugs to save lives by inhibiting kinases in cancer cells, they may be inadvertently triggering a death program in healthy erythrocytes.

The mechanisms underpinning kinase inhibitor-induced eryptosis are multifaceted and appear to converge on the classical Ca^2+^-centric pathway, though the precise upstream events remain a subject of intense study. Some inhibitors may directly or indirectly activate the Ca^2+^-permeable TRPC6 channels [6]. Others may deplete intracellular ATP, a critical cofactor for the flippase and for maintaining ion gradients, thereby sensitizing the cell to eryptosis [26]. A significant number of these agents are also known to induce oxidative stress, generating ROS that further destabilize the erythrocyte membrane and potentiate Ca^2+^ entry [27]. Furthermore, many kinase inhibitors target the phosphoinositide 3-kinase (PI3K)/Akt and other survival pathways. While erythrocytes lack the transcriptional machinery for classic apoptosis, these pathways can influence the activity of kinases like protein kinase C (PKC) and the adenosine monophosphate-activated protein kinase (AMPK), which can modulate ion channel activity and the eryptotic machinery [28,29,30]. The net effect is a drug-induced acceleration of the erythrocyte’s intrinsic aging and death program.

The clinical implications of this phenomenon are potentially significant, though they remain to be fully established in clinical settings. From a safety pharmacology perspective, the eryptotic potential of a kinase inhibitor constitutes a mechanistically plausible off-target toxicity that may contributes to anemia, reduces patient quality of life, and could necessitate dose reductions or treatment interruptions that compromise therapeutic efficacy [31,32]. The chronic loss of RBCs can also lead to compensatory reticulocytosis, which, while maintaining hemoglobin levels, increases the burden on the bone marrow and can lead to the circulation of younger, potentially more vulnerable RBCs [33]. From a pathophysiological standpoint, the accelerated clearance of eryptotic erythrocytes may places a burden on the phagocytic system, particularly the splenic macrophages [34]. The release of microvesicles from eryptotic cells has been proposed to contribute to a pro-thrombotic state, as these vesicles often carry PS and tissue factor; however, whether drug-induced eryptosis directly increases thromboembolic risk in patients receiving TKIs remains to be demonstrated [35,36,37]. Furthermore, the systemic clearance of PS-exposing RBCs could theoretically represent a chronic, low-grade inflammatory stimulus, which might contributing to the fatigue and other systemic symptoms experienced by patients on long-term therapy although this hypothesis requires prospective clinical validation [38].

This review provides a comprehensive and critical analysis of the current state of knowledge regarding the induction of eryptosis by pharmacological modulators of the phosphorylation balance. We here systematically evaluate the diverse classes of kinase inhibitors, from first-generation TKIs to novel selective agents, as well as the emerging class of phosphatase inhibitors, examining the evidence for their eryptotic potential and the proposed molecular mechanisms driving this effect. A particular focus is placed on dissecting whether this phenomenon represents a common off-target liability or one that is specific to certain chemical scaffolds and molecular targets. Also, we synthesize the clinical implications, discussing the impact on patient management, the potential for using eryptotic biomarkers as predictive tools for drug-induced anemia, and the broader pathophysiological consequences of chronic, drug-accelerated erythrocyte clearance. By integrating current mechanistic insights with clinical observations, this review underscores the importance of considering the kinase–phosphatase equilibrium not just as a regulator of intracellular signaling, but as a critical determinant of drug safety and efficacy in the era of targeted cancer therapy.

## 2. Pharmacological Modulators of the Kinase–Phosphatase Equilibrium as Inducers of Eryptosis

A growing body of evidence has identified that a diverse array of pharmacological inhibitors, targeting both kinases and their counter-regulatory phosphatases, inadvertently trigger the suicidal death of human erythrocytes. This section systematically reviews these compounds, detailing their primary molecular targets and the mechanisms by which they induce eryptosis. The hallmarks of this process, including PS exposure, cell shrinkage, Ca^2+^ influx, oxidative stress, and ceramide accumulation serve as the readout for the pro-eryptotic activity of these drugs.

It is important to note that most of the evidence presented in this section is derived from in vitro experiments using erythrocytes from healthy donors. These studies provide robust mechanistic insights, but they do not fully recapitulate the complex pharmacokinetic, metabolic, and physiological conditions present in patients. We have therefore indicated, for each compound, the experimental system used and whether the evidence constitutes direct demonstration of eryptosis in mature human erythrocytes or indirect mechanistic support derived from other cellular systems.

### 2.1. Inhibitors Targeting Cell Cycle and Mitotic Kinases

Several inhibitors designed to arrest the cell cycle in nucleated cancer cells have demonstrated unexpected effects on anucleate erythrocytes. *BI2536*, a potent inhibitor of polo-like kinases (PLK1, PLK2, PLK3), which are critical regulators of mitosis, induced eryptosis in human erythrocytes after 48 h of exposure [39]. The eryptotic phenotype was characterized by significant cell shrinkage (decreased forward scatter) and increased PS exposure (annexin-V binding), without evidence of hemolysis. Mechanistically, the effect was linked to a significant increase in cytosolic Ca^2+^ concentration and an abundance of ceramide, suggesting that BI2536 appears to engage the classical Ca^2+^-dependent pathway of eryptosis despite the absence of a nucleus. BI2536 is a unique compound so far in the literature that illustrates the mechanistic principle that cell cycle kinase inhibitors can affect anucleate erythrocytes.

### 2.2. Tyrosine Kinase Inhibitors (TKIs)

Tyrosine kinase inhibitors represent one of the most extensively studied classes of pharmacological kinase inhibitors in the context of eryptosis. These agents, developed primarily for the treatment of chronic myeloid leukemia, non-small cell lung cancer, and other malignancies, consistently trigger suicidal erythrocyte death through diverse molecular mechanisms.

***Dasatinib***, a second-generation BCR-ABL and Src family kinase inhibitor, was among the first TKIs identified as a potent eryptosis inducer. Chan et al. (2018) demonstrated that dasatinib-treated human erythrocytes exhibited hallmark features of eryptosis, including cell shrinkage, loss of membrane integrity, and PS exposure [25]. Mechanistically, the increase in cytosolic Ca^2+^ concentration was identified as a key mediator, as depletion of extracellular Ca^2+^ significantly suppressed the eryptotic effects. Additionally, dasatinib induced early denaturation of esterase, caspase-3 activation, and alterations in cellular redox state, with membrane permeabilization occurring in a manner similar to digitonin. The study concluded that dasatinib triggers eryptosis through Ca^2+^ loading and direct membrane permeabilization.

***Sunitinib***, a multi-kinase inhibitor targeting VEGFR, PDGFR, and other receptors, was similarly shown to induce eryptosis. Shaik et al. (2012) reported that exposure to sunitinib for 48 h resulted in significant cell shrinkage and PS exposure, accompanied by a modest increase in hemolysis [40]. The eryptotic effect was partially dependent on Ca^2+^ influx and was significantly blunted by the p38 MAPK inhibitor SB203580 and the pancaspase inhibitor zVAD. Notably, sunitinib did not alter cytosolic ATP levels or ceramide abundance, suggesting that its pro-eryptotic action is mediated through Ca^2+^-dependent activation of p38 kinase and caspases rather than through metabolic depletion or sphingolipid signaling.

***Afatinib***, an irreversible EGFR and HER2 inhibitor used in non-small cell lung cancer, also stimulates eryptosis. Bhuyan and Lang (2018) demonstrated that afatinib-treated erythrocytes exhibited increased PS exposure, decreased forward scatter (cell shrinkage), and elevated cytosolic Ca^2+^ levels [41]. The effect was accompanied by increased reactive oxygen species (ROS) generation and ceramide accumulation at the erythrocyte surface. The dependence on Ca^2+^ was confirmed by the observation that removal of extracellular Ca^2+^ significantly blunted the eryptotic response. Thus, afatinib triggers phospholipid scrambling through a mechanism involving Ca^2+^ entry, oxidative stress, and ceramide formation.

***Gefitinib***, another EGFR tyrosine kinase inhibitor, shares a similar pro-eryptotic profile. Bhuyan et al. (2017) reported that exposure to gefitinib for 48 h induced cell shrinkage and PS exposure in a concentration-dependent manner [42]. Although gefitinib did not significantly increase Fluo3 fluorescence (a measure of cytosolic Ca^2+^), the effect on PS exposure was nonetheless dependent on extracellular Ca^2+^, as removal of Ca^2+^ from the medium significantly blunted the response. Furthermore, gefitinib treatment significantly increased DCFDA fluorescence, indicating ROS accumulation. These findings suggest that gefitinib-induced eryptosis involves both Ca^2+^ entry and oxidative stress.

***Lapatinib***, a dual EGFR/HER2 inhibitor, presents a more complex mechanism. Zierle et al. (2015) found that lapatinib significantly increased PS exposure and cell shrinkage [43]. Surprisingly, lapatinib treatment resulted in a slight but significant decrease in cytosolic Ca^2+^ concentration, ruling out Ca^2+^ influx as a triggering mechanism. The effect on PS exposure was not blunted by Ca^2+^ removal, and lapatinib did not significantly modify ROS or ceramide levels. Notably, lapatinib enhanced the eryptotic effect of the Ca^2+^ ionophore ionomycin without further increasing Ca^2+^ levels. The authors concluded that lapatinib triggers eryptosis through a Ca^2+^-independent mechanism, distinguishing it from other TKIs that rely on Ca^2+^ influx.

***Ceritinib***, an ALK inhibitor used in ALK-positive non-small cell lung cancer, induces eryptosis through a multi-kinase pathway. Bhuyan et al. (2016) demonstrated that ceritinib treatment resulted in PS exposure, cell shrinkage, and increased cytosolic Ca^2+^ levels [44]. The eryptotic effect was significantly blunted by removal of extracellular Ca^2+^, as well as by the kinase inhibitors staurosporine (PKC inhibitor), SB203580 (p38 MAPK inhibitor), and D4476 (CK1α inhibitor). Additionally, the pancaspase inhibitor zVAD significantly attenuated the response, indicating that caspase activation also contributes. These findings establish that ceritinib triggers eryptosis through a coordinated mechanism involving Ca^2+^ entry and the activation of multiple kinases and caspases.

***Ruxolitinib***, a JAK1/JAK2 inhibitor used in myeloproliferative neoplasms, induces eryptosis through a predominantly p38 MAPK-dependent pathway. Briglia et al. (2015) reported that ruxolitinib treatment increased PS exposure and decreased cell volume without significant changes in cytosolic Ca^2+^ or ROS levels [45]. The eryptotic effect was virtually abolished by the p38 MAPK inhibitors SB203580 and skepinone, demonstrating that p38 MAPK activity is required for ruxolitinib-induced erythrocyte death. Whether p38 activation alone is sufficient to trigger this effect remains to be established, as pharmacological inhibition only addresses necessity.

***Pazopanib***, a multi-targeted kinase inhibitor (VEGFR, PDGFR, c-Kit), also stimulates eryptosis through a combination of pathways. Signoretto et al. (2016) demonstrated that pazopanib treatment induced PS exposure, cell shrinkage, and hemolysis in human erythrocytes [46]. The effect was partially dependent on extracellular Ca^2+^, as removal of Ca^2+^ significantly blunted but did not abolish PS exposure. Pazopanib treatment also significantly increased DCFDA fluorescence (ROS generation) and ceramide abundance at the erythrocyte surface. Thus, pazopanib-induced eryptosis involves the convergence of Ca^2+^ entry, oxidative stress, and ceramide accumulation.

***Sorafenib***, a multi-kinase inhibitor targeting Raf, VEGFR, and PDGFR, has been studied both in vitro and in vivo. Lupescu et al. (2012) showed that sorafenib treatment for 48 h significantly increased cytosolic Ca^2+^, decreased cell volume, and induced PS exposure with slight hemolysis [27]. The effect was associated with increased DCFDA fluorescence, indicating oxidative stress, and the reducing agents N-acetylcysteine and tiron significantly blunted sorafenib-induced PS exposure. Notably, sorafenib did not alter ceramide abundance or cytosolic ATP levels. Importantly, the study extended to in vivo findings: hepatocellular carcinoma patients undergoing Sorafenib chemotherapy exhibited a significant increase in circulating PS-exposing erythrocytes, confirming the clinical relevance of these findings.

JAK3, a tyrosine kinase expressed in hematopoietic tissues, has been identified as a pro-eryptotic mediator. Bhavsar et al. (2011) demonstrated that JAK3 is expressed in human erythrocytes and becomes phosphorylated upon energy depletion [47]. Glucose withdrawal increased PS exposure, an effect significantly blunted in erythrocytes from JAK3-deficient mice and in the presence of ***JAK3 inhibitors***. These findings establish JAK3 as a critical regulator of eryptosis under conditions of metabolic stress.

Collectively, the data on TKIs reveal a spectrum of pro-eryptotic mechanisms. While many TKIs converge on Ca^2+^ influx as a central mediator (dasatinib, sunitinib, afatinib, gefitinib, ceritinib, pazopanib, sorafenib), others operate through Ca^2+^-independent pathways (lapatinib, ruxolitinib). Downstream signaling often involves p38 MAPK activation, PKC activation, caspase activation, and oxidative stress, with ceramide accumulation playing a contributory role in some cases. The diversity of mechanisms underscores that the eryptotic potential of TKIs is not a single uniform phenomenon but rather a complex outcome of target-specific and off-target effects on erythrocyte signaling networks.

### 2.3. Inhibitors of the PI3K/AKT/mTOR Signaling Axis

The PI3K/AKT/mTOR signaling axis represents a central pathway governing cell survival, growth, and metabolism in nucleated cells. Pharmacological inhibitors targeting this cascade have become cornerstone agents in oncology, yet their effects on anucleate erythrocytes reveal unexpected and complex pro-eryptotic activities.

***LY294002***, a potent and selective inhibitor of phosphoinositide 3-kinase (PI3K), has been shown to induce apoptosis in human erythroid progenitor cells. Haseyama et al. (1999) demonstrated that LY294002 dose-dependently inhibits the proliferation of erythroid colony-forming cells and induces apoptosis, as evidenced by annexin-V binding and DNA fragmentation [48]. This effect was observed even in the presence of erythropoietin (EPO), suggesting that PI3K activity is essential for erythroid cell survival independently of EPO signaling. Paiboonsukwong et al. (2003) further showed that LY294002 at concentrations as low as 10 µM abolishes the anti-apoptotic effect of EPO in erythroid progenitor cells, while not affecting the survival signals mediated by interferon-γ [49]. The mechanism involves suppression of Bcl-x expression and blockade of Akt phosphorylation. These findings establish that PI3K inhibition removes a critical pro-survival brake, thereby sensitizing erythroid cells to apoptotic and eryptotic stimuli. Importantly, these studies were performed in erythroid progenitor cells and thus provide indirect evidence for a role of PI3K in erythrocyte survival, rather than direct demonstration of eryptosis in mature erythrocytes.

***Wortmannin***, another irreversible PI3K inhibitor, similarly sensitizes erythroid cells to apoptosis. Bavelloni et al. (2000) showed that wortmannin impairs pre-commitment cell cycle traverse and prevents differentiation in erythroleukemia cells [50]. By blocking PI3K/Akt signaling, wortmannin promotes cell death under conditions that normally support survival. As with LY294002, these findings in erythroleukemia cells provide indirect mechanistic support rather than direct evidence of eryptosis in mature erythrocytes.

***MK-2206***, an allosteric pan-Akt inhibitor, represents a more selective tool for targeting the PI3K/Akt axis at the level of Akt itself. Simioni et al. (2013) demonstrated that MK-2206 induces apoptosis in cancer cells characterized by hyperphosphorylated Akt-1, an effect mediated by downregulation of GSK3α/β and FOXO3A phosphorylation [51]. Ramakrishnan et al. (2012) further showed that MK-2206 induces cytotoxicity in multiple myeloma cells through caspase-dependent apoptosis and inhibits proliferation even in the presence of bone marrow stromal cells [52]. While direct studies on mature erythrocytes are limited, the established role of Akt as a pro-survival kinase in erythroid cells strongly suggests that MK-2206 would similarly induce eryptosis by blocking this protective pathway. However, direct evidence in mature erythrocytes is currently lacking, and this remains a hypothesis requiring experimental validation.

***Temsirolimus***, an mTOR inhibitor widely used in renal cell carcinoma and other malignancies, has been demonstrated to induce eryptosis through multiple converging pathways in mature human erythrocytes. Bhuyan et al. (2017) reported that exposure of human erythrocytes to temsirolimus for 48 h resulted in significant cell shrinkage and increased PS exposure at the erythrocyte surface [53]. The pro-eryptotic effect was accompanied by a significant increase in cytosolic Ca^2+^ concentration, ROS generation, and ceramide abundance. Importantly, the effect on PS exposure was partially dependent on extracellular Ca^2+^, as removal of Ca^2+^ from the medium significantly blunted the response. Pharmacological dissection of the downstream signaling revealed that the effect was significantly reduced by staurosporine and chelerythrine, both inhibitors of protein kinase C (PKC), indicating that PKC activation plays a critical role. In contrast, the p38 MAPK inhibitor SB203580, the casein kinase 1α (CK1α) inhibitor D4476, and the pancaspase inhibitor zVAD did not significantly modify temsirolimus-induced PS exposure, distinguishing its mechanism from that of other kinase inhibitors. Notably, chelerythrine also significantly blunted temsirolimus-induced ROS formation, suggesting that PKC activation occurs upstream of oxidative stress in this context. The authors concluded that temsirolimus triggers eryptosis through a pathway involving Ca^2+^ entry, oxidative stress, ceramide accumulation, and activation of staurosporine- and chelerythrine-sensitive kinases, primarily PKC.

***Cepharanthine***, a bisbenzylisoquinoline alkaloid that functions as an inhibitor of PI3K, AKT, mTOR, and AMPK, represents a mechanistically distinct example within this class and is an experimental compound. Alruwaili et al. (2025) provided the first comprehensive investigation of cepharanthine’s cytotoxicity to human erythrocytes, revealing a remarkably unique pro-eryptotic mechanism [54]. Exposure to cepharanthine induced concentration-dependent PS exposure, cell shrinkage, hemolysis, and prominent membrane blebbing in mature human erythrocytes. However, in a striking departure from most other eryptosis inducers, cepharanthine did not elevate cytosolic Ca^2+^ levels. The effect was entirely Ca^2+^-independent, as demonstrated by the absence of Fluo4 fluorescence increase and the failure of Ca^2+^ removal to blunt the eryptotic response. Mechanistic dissection using pharmacological inhibitors revealed a complex signaling network: the p38 MAPK inhibitor SB203580, the CK1α inhibitor D4476, acetylsalicylic acid (a COX inhibitor), necrosulfonamide (an MLKL inhibitor), and melatonin all significantly inhibited both PS exposure and hemolysis. In contrast, staurosporine (PKC inhibitor), L-NAME (iNOS inhibitor), ascorbate, caffeine, adenine, and guanosine only prevented hemolysis without affecting PS exposure. Interestingly, sucrose had a dual effect, exacerbating PS exposure while reversing hemolysis. The study also revealed that chelation of intracellular Ca^2+^ with BAPTA-AM reduced hemolysis, indicating that while extracellular Ca^2+^ is not required, basal intracellular Ca^2+^ may still play a permissive role. The authors concluded that cepharanthine activates Ca^2+^-independent pathways to promote eryptosis and hemolysis through a complex signaling axis involving p38 MAPK, CK1α, COX, MLKL, PKC, and iNOS, with metabolic insufficiency and dysregulated ion trafficking serving as key contributors.

The contrasting profiles of temsirolimus and cepharanthine highlight the diversity within the PI3K/AKT/mTOR inhibitor class. The inhibition of the PI3K/AKT/mTOR axis can trigger erythrocyte death through distinct mechanisms depending on the specific inhibitor, its off-target effects, and the cellular context. Moreover, the Ca^2+^-independent nature of cepharanthine-induced eryptosis represents a paradigm shift in understanding how kinase inhibitors can trigger suicidal erythrocyte death without engaging the classical Ca^2+^-centric pathway.

### 2.4. Modulators of MAPK Pathways and Casein Kinases

The mitogen-activated protein kinase (MAPK) pathways, particularly p38 MAPK, and casein kinases (CK1α) have emerged as critical downstream signaling hubs in the execution of eryptosis. Pharmacological modulators targeting these kinases, whether through direct activation or indirect modulation, have provided invaluable insights into the molecular machinery governing suicidal erythrocyte death. Unlike the tyrosine kinase inhibitors discussed previously, which primarily target receptors or upstream signaling molecules, the compounds in this section act directly on the kinases that orchestrate the terminal phases of the eryptotic program.

***Pyrvinium pamoate***, an experimental compound and a specific activator of casein kinase 1α (CK1α), has been instrumental in establishing the role of this kinase in eryptosis regulation. Kucherenko et al. (2012) investigated the effects of pyrvinium pamoate on erythrocyte ion channels and cell death [55]. Their study revealed that CK1α activation significantly augmented the eryptotic response to Cl^−^ removal, a well-established trigger of Ca^2+^ influx and cell membrane scrambling. Specifically, pyrvinium pamoate enhanced the increase in cytosolic Ca^2+^ concentration and PS exposure induced by Cl^−^ removal, while paradoxically blunting the cell shrinkage typically associated with these events. Whole-cell patch clamp recordings confirmed that pyrvinium pamoate activated Cl^−^-sensitive Ca^2+^-permeable cation channels, leading to enhanced Ca^2+^ entry, while simultaneously inhibiting Ca^2+^-activated K^+^ channels (Gardos channels), thereby limiting K^+^ efflux and subsequent cell shrinkage. This dual effect dissociates the two cardinal hallmarks of eryptosis, the PS exposure and cell shrinkage, demonstrating that they can be independently regulated and that CK1α sits at a critical nodal point controlling both ion channel activity and membrane scrambling.

PAK2 (p21-activated kinase 2) has been identified as another critical regulator of eryptosis. Zelenak et al. (2011) performed a proteome analysis of erythrocytes lacking AMP-activated protein kinase (AMPK) and identified PAK2 as downregulated [56]. Specific inactivation of PAK2 with the inhibitor *IPA3* (experimental compound) in human erythrocytes increased PS exposure and augmented the stimulating effect of glucose deprivation on annexin-V-binding. These findings established PAK2 as a kinase whose activity is required for erythrocyte survival under stress conditions, positioning it as a negative regulator of eryptosis rather than a pro-eryptotic kinase. Inactivation of PAK2 sensitizes erythrocytes to eryptotic stimuli, indicating that its physiological role is cytoprotective under metabolic stress conditions.

The p38 MAPK pathway, a key mediator of eryptosis, has been extensively characterized using selective pharmacological inhibitors. Gatidis et al. (2011) demonstrated that p38 kinase is expressed in human erythrocytes and becomes phosphorylated (activated) upon hyperosmotic shock [57]. The p38 inhibitors SB203580 and p38 Inh III significantly blunted the eryptotic effects of hyperosmotic stress, including the decrease in cell volume, the increase in PS exposure, and the elevation of cytosolic Ca^2+^. These findings established that p38 MAPK activation is required for eryptosis under conditions of osmotic stress, positioning it downstream of Ca^2+^ entry and upstream of the membrane scrambling machinery. However, as these data rely on pharmacological inhibition alone, they demonstrate necessity rather than sufficiency, and gain-of-function studies would be required to establish whether p38 activation is sufficient to trigger eryptosis.

Collectively, the studies on MAPK pathway and casein kinase modulators reveal a hierarchical signaling network in which CK1α, PAK2, and p38 MAPK serve as critical executioners of the eryptotic program. The ability to induce eryptosis via CK1α activation with pyrvinium pamoate and to identify PAK2 as a pro-eryptotic kinase underscores the importance of these pathways in drug-induced erythrocyte death.

### 2.5. Inhibitors of Spleen Tyrosine Kinase (SYK) and Janus Kinase 2 (JAK2)

***R406***, the active metabolite of the prodrug ***fostamatinib***, is a potent inhibitor of spleen tyrosine kinase (SYK). Fostamatinib is approved for the treatment of chronic immune thrombocytopenia (ITP) and acts by inhibiting Fc receptor-triggered, SYK-dependent cytoskeletal rearrangement during phagocytosis [58]. While the primary clinical effect is to reduce platelet destruction, SYK is also expressed in erythrocytes and may play a role in erythrocyte survival. Parachalil Gopalan et al. (2026) demonstrated that SYK inhibition can sensitize erythrocytes to eryptotic stimuli, and modulation of SYK activity may contribute to the anemia observed in some patients treated with fostamatinib [59]. Matsukane et al. (2022) further characterized the pharmacokinetics and pharmacodynamics of fostamatinib and its active moiety R406, confirming its potent SYK inhibitory activity [60].

***AG490***, a pharmacological inhibitor of Janus kinase 2 (JAK2) and experimental compound, has been extensively studied in hematopoietic cells. Nagao et al. (2011) demonstrated that AG490 induces degradation of both wild-type JAK2 and the constitutively active JAK2-V617F mutant in hematopoietic cells, leading to synergistic induction of apoptosis when combined with DNA-damaging agents [61]. This effect is mediated through activation of GSK3β and subsequent proteasomal degradation of JAK2. Sandberg et al. (2004) further showed that AG490 blocks JAK2-dependent induction of MKP-1, thereby altering MAPK signaling dynamics [62]. Given that JAK2 is expressed in erythrocytes and becomes phosphorylated upon energy depletion, JAK2 inhibition may promote eryptosis under conditions of metabolic stress. However, direct evidence of eryptosis in mature erythrocytes for AG490 is limited.

### 2.6. Inhibitors of AMP-Activated Protein Kinase (AMPK)

***Dorsomorphin***, a selective inhibitor of AMP-activated protein kinase (AMPK), and also an experimental compound, provides insight into the role of this energy-sensing kinase in erythrocyte survival. AMPK acts as a cellular energy sensor that counteracts energy depletion by stimulating energy production and limiting energy utilization. Föller et al. (2009) demonstrated that AMPK is expressed in erythrocytes and that its activation protects against eryptosis [63]. Conversely, inhibition of AMPK with Compound C sensitizes erythrocytes to energy depletion-induced cell death. The AMPK activator AICAR has been shown to blunt eryptosis triggered by glucose withdrawal, confirming that AMPK activity promotes erythrocyte survival. Therefore, pharmacological inhibition of AMPK represents a pro-eryptotic mechanism that operates through disruption of cellular energy homeostasis.

### 2.7. Natural Products and Other Kinase Modulators

A significant and growing body of evidence has identified numerous natural products with anticancer activity as potent inducers of eryptosis. These compounds, derived from plants, fungi, and marine organisms, often function as kinase inhibitors or modulators and have been extensively studied for their cytotoxic effects on human erythrocytes. Their mechanisms are remarkably diverse, spanning the full spectrum of eryptotic signaling pathways. Many of these natural products are experimental compounds not currently approved for clinical use, but they are included because they provide valuable mechanistic insights into the regulation of eryptosis.

***Thioridazine***, a neuroleptic phenothiazine with antimicrobial and anticancer properties, was among the first natural product-derived compounds shown to induce eryptosis through kinase-dependent mechanisms. Lang et al. (2013) demonstrated that exposure to thioridazine for 48 h resulted in significant PS exposure, cell shrinkage, and increased cytosolic Ca^2+^ concentration in human mature erythrocytes [64]. The effect was partially dependent on extracellular Ca^2+^ and was significantly blunted by the p38 MAPK inhibitor SB203580, indicating that p38 kinase activation downstream of Ca^2+^ influx contributes to the eryptotic response. Thioridazine is now recognized as a direct inhibitor of PI3K, AKT, and mTOR, positioning it as a bona fide kinase inhibitor whose pro-eryptotic activity aligns with the broader theme of kinase inhibitor-induced erythrocyte death.

***Juglone***, a naphthoquinone isolated from Juglans mandshurica Maxim, exhibits potent anticancer activity and has been shown to trigger eryptosis through a mechanism distinct from thioridazine. Calabrò et al. (2015) reported that juglone treatment induced PS exposure, cell shrinkage, increased ceramide abundance, and ATP depletion in human mature erythrocytes [65]. The effect was partially dependent on extracellular Ca^2+^ and was significantly blunted by the PKC inhibitor staurosporine, indicating that PKC activation plays a critical role. Notably, juglone did not significantly alter ROS levels, suggesting that its pro-eryptotic action is mediated primarily through ceramide upregulation, energy depletion, and PKC activation rather than oxidative stress. Juglone directly inhibits PI3K and AKT, further establishing the link between kinase inhibition and eryptosis.

***Boswellic acid***, a pentacyclic triterpene derived from Boswellia serrata gum resin, has been investigated for its eryptotic potential by Calabrò et al. (2015) [66]. Exposure to boswellic acid for 24 h resulted in significant PS exposure and cell shrinkage in human mature erythrocytes without affecting cytosolic Ca^2+^, ATP, ROS, or ceramide levels [66]. The effect was significantly blunted by the p38 MAPK inhibitors skepinone and SB203580, indicating that p38 MAPK activity is required for boswellic acid-induced eryptosis. However, as these data rely on pharmacological inhibition alone, they demonstrate necessity rather than sufficiency; gain-of-function studies would be required to establish whether p38 activation alone is sufficient to trigger the eryptotic response. This Ca^2+^-independent mechanism distinguishes boswellic acid from many other natural products and positions p38 MAPK as a critical downstream effector even in the absence of Ca^2+^ signaling. Boswellic acid directly inhibits IKK, PI3K, AKT, and mTOR, confirming its status as a multi-kinase inhibitor.

***Fucoxanthin***, a carotenoid isolated from brown seaweed, induces eryptosis through a predominantly Ca^2+^-dependent mechanism in human mature erythrocytes. Briglia et al. (2015) demonstrated that fucoxanthin treatment resulted in PS exposure, cell shrinkage, hemolysis, increased cytosolic Ca^2+^, and lipid peroxidation, without significant changes in ROS levels [67]. The effect was partially blunted by removal of extracellular Ca^2+^ but was insensitive to p38 MAPK and PKC inhibitors, suggesting that fucoxanthin acts directly on Ca^2+^-permeable channels or the membrane itself to trigger phospholipid scrambling. Fucoxanthin directly inhibits PI3K, AKT, and mTOR, yet its eryptotic mechanism appears to bypass the classical kinase-dependent pathways activated by other PI3K/AKT/mTOR inhibitors.

***Gingerol ([6]-gingerol)***, the major polyphenol in Zingiber officinale, has been shown to induce eryptosis through a unique mechanism involving both Ca^2+^ influx and CK1α activation in human mature erythrocytes. Alamri et al. (2020) reported that gingerol treatment induced significant hemolysis at high concentrations, along with PS exposure, cell shrinkage, and elevated cytosolic Ca^2+^ [68]. The eryptotic effect was thoroughly abrogated by removal of extracellular Ca^2+^, confirming Ca^2+^ dependence. Importantly, the CK1α inhibitor D4476 significantly attenuated gingerol-induced hemolysis, demonstrating that CK1α activation is required for the toxic effect. Gingerol inhibits PI3K, AKT, and mTOR while activating CK1α, representing a dual mechanism that combines kinase inhibition with kinase activation to trigger erythrocyte death.

***Arctigenin***, a lignan with promising anticancer activity, has been shown to induce Ca^2+^-dependent eryptosis through energy depletion and CK1α activation. Alfhili et al. (2025) demonstrated that arctigenin treatment increased PS exposure and cell shrinkage, effects that were inhibited by extracellular Ca^2+^ removal [69]. Suppression of hemolysis required both Ca^2+^ exclusion and restriction of K^+^ efflux, indicating dysregulated ion trafficking. Notably, sucrose and mannitol rescued cells from hemolysis while paradoxically exacerbating PS exposure, which was significantly blunted by guanosine and the CK1α inhibitor D4476. The authors concluded that arctigenin promotes Ca^2+^-dependent eryptosis through metabolic collapse and CK1α activation.

***Eriocitrin***, a flavanone glycoside in citrus fruits, induces eryptosis through a combination of Ca^2+^ accumulation, oxidative stress, and activation of CK1α and Rac1 GTPase in human mature erythrocytes. Alghareeb et al. (2023) reported that eriocitrin treatment resulted in concentration-dependent hemolysis, PS exposure, Ca^2+^ elevation, and oxidative stress [70]. The hemolytic activity was significantly ameliorated by the CK1α inhibitor D4476 and the Rac1 inhibitor NSC23766, confirming the involvement of these signaling mediators. Additionally, isosmotic urea, sucrose, and polyethylene glycol 8000 protected against hemolysis, implicating osmotic stress in the mechanism.

***Galangin***, a flavonoid with anticancer properties, triggers eryptosis and hemolysis through a complex signaling axis involving PKC, CK1α, COX, p38, and Rac1 in human mature erythrocytes. Alfhili et al. (2024) demonstrated that galangin induced PS exposure, cell shrinkage, Ca^2+^ accumulation, and reduced AChE activity, with associated formation of granulated echinocytes [71]. The effect was blunted by Ca^2+^ omission and energy replenishment with glucose, adenine, and guanosine. Co-treatment with staurosporine (PKC inhibitor), D4476 (CK1α inhibitor), or acetylsalicylic acid (COX inhibitor) prevented PS externalization, whereas only SB203580 (p38 inhibitor) and NSC23766 (Rac1 inhibitor) rescued cells from hemolysis. The authors concluded that galangin-induced eryptosis is driven by Ca^2+^ nucleation and metabolic collapse mediated by the PKC/CK1α/COX/p38/Rac1 signaling axis.

***Deguelin***, a retinoid isolated from various plants, induces eryptosis through a Ca^2+^-independent and ROS-independent mechanism involving CK1α activation and ATP depletion in human mature erythrocytes. Alfhili and Alsughayyir (2023) reported that deguelin treatment resulted in PS exposure and cell shrinkage without Ca^2+^ elevation or oxidative stress [72]. The effect was ameliorated by preventing KCl efflux and in the presence of sucrose, D4476, and ATP. Notably, deguelin also significantly reduced reticulocyte count and increased platelet distribution width in whole blood, indicating specific toxicity towards young erythrocytes and platelets.

***Costunolide***, a natural sesquiterpene lactone, induces eryptosis through PKC-α activation coupled with glutathione depletion and G6PDH inhibition in human mature erythrocytes. Ghashghaeinia et al. (2020) demonstrated that costunolide treatment enhanced PS exposure, decreased cell volume, depleted intracellular glutathione, and completely inhibited G6PDH activity [73]. The specific PKC-α inhibitor chelerythrine significantly reversed the effects on PS exposure and cell volume without affecting glutathione depletion, indicating that PKC-α activation is a critical mediator of costunolide-induced eryptosis independent of the oxidative stress pathway.

***Sclareol***, a diterpene alcohol with proposed anticancer activity, induces eryptosis through p38 MAPK and CK1α activation in a Ca^2+^-independent manner in human mature erythrocytes. Signoretto et al. (2016) reported that sclareol treatment increased PS exposure and hemolysis without significantly altering cell volume, ROS, or ceramide [74]. Although sclareol increased Fluo3 fluorescence, removal of extracellular Ca^2+^ did not blunt the PS exposure effect. Instead, the effect was significantly reduced by the p38 MAPK inhibitor skepinone and the CK1α inhibitor D4476, confirming that these kinases are required for sclareol-induced eryptosis.

***Embelin***, a phytochemical with antihelminthic and anticancer properties, induces eryptosis through ceramide accumulation independent of Ca^2+^, p38, and PKC in human mature erythrocytes. Bouguerra et al. (2015) demonstrated that embelin treatment increased PS exposure and hemolysis without modifying cytosolic Ca^2+^ [75]. The effect was not blunted by removal of extracellular Ca^2+^, by p38 inhibitor SB203580, or by PKC inhibitor staurosporine, but was associated with increased ceramide abundance, identifying ceramide as the primary mediator.

***Phloretin***, a natural component of apples, pears, and strawberries, similarly induces PS exposure through ceramide accumulation without involving Ca^2+^, ATP, PKC, or p38 in human mature erythrocytes. Bissinger et al. (2014) reported that phloretin treatment increased PS exposure without affecting cell volume, cytosolic Ca^2+^, or ATP levels, and the effect was not modified by Ca^2+^ removal, staurosporine, or SB203580, but was associated with increased ceramide abundance [76].

***Manumycin A***, a streptomycete-derived farnesyltransferase inhibitor, triggers eryptosis through Ca^2+^ influx and activation of PKC and p38 MAPK in human mature erythrocytes. Egler et al. (2016) demonstrated that manumycin A treatment induced PS exposure, cell shrinkage, and hemolysis [77]. The effect was significantly blunted by removal of extracellular Ca^2+^, by staurosporine, and by SB203580, indicating that Ca^2+^ entry and activation of PKC and p38 MAPK collectively contribute to the eryptotic response.

***Camalexin***, a phytoalexin from Arabidopsis thaliana, induces eryptosis through Ca^2+^ influx, PKC activation, and caspase activation in human mature erythrocytes. Almasry et al. (2017) reported that camalexin treatment increased PS exposure, cell shrinkage, and cytosolic Ca^2+^ without affecting ROS or ceramide [78]. The effect was blunted by removal of extracellular Ca^2+^, by staurosporine and chelerythrine, and by the caspase inhibitors zVAD and zIETD-fmk, identifying a coordinated pathway involving Ca^2+^, PKC, and caspases.

***β-Caryophyllene oxide***, a sesquiterpene with anticancer properties, induces hemolysis and eryptosis through energy depletion, Ca^2+^ accumulation, oxidative stress, and activation of CK1α, PKC, and MLKL in human mature erythrocytes. Alghareeb et al. (2023) demonstrated that β-caryophyllene oxide treatment induced PS exposure, cell shrinkage, Ca^2+^ accumulation, oxidative stress, and reduced AChE activity [79]. The toxicity was ameliorated by the CK1α inhibitor D4476, the PKC inhibitor staurosporine, and the MLKL inhibitor necrosulfonamide, establishing a complex signaling network involving these mediators.

***α-Linolenic acid***, an omega-3 fatty acid with anticancer properties, stimulates hemolysis and eryptosis through Ca^2+^ buildup, oxidative stress, anticholinesterase activity, CK1α activation, and MLKL activation in human mature erythrocytes. Alharthy et al. (2024) reported that α-linolenic acid treatment increased hemolysis and PS exposure in a concentration-dependent manner, along with elevated Ca^2+^ and ROS, and reduced AChE activity [80]. The addition of D4476 and necrosulfonamide significantly inhibited α-linolenic acid toxicity, confirming the involvement of CK1α and MLKL.

To summarize, the natural products discussed in this section reveal a remarkable diversity of mechanisms by which kinase-modulating compounds can induce eryptosis. While many converge on Ca^2+^ influx and activation of p38 MAPK, PKC, or CK1α, others operate through ceramide accumulation, ATP depletion, or oxidative stress independently of Ca^2+^. The complex signaling networks identified, ranging from the PKC/CK1α/COX/p38/Rac1 axis in galangin to the p38/CK1α/COX/MLKL/PKC/iNOS network in cepharanthine, highlight that natural products often engage multiple pathways simultaneously. These findings carry important implications for the clinical development of natural product-based anticancer therapies, as the eryptotic potential of these compounds may contribute to chemotherapy-induced anemia while also offering opportunities for therapeutic intervention using eryptosis inhibitors, Ca^2+^ channel blockers, and antioxidants.

### 2.8. Phosphatase Inhibitors as Inducers of Eryptosis via Kinase Activation

While the preceding sections have focused on compounds that directly inhibit kinases, a parallel and equally important class of eryptosis inducers comprises inhibitors of serine/threonine protein phosphatases. These agents, by blocking the enzymes that remove phosphate groups from proteins, shift the cellular phosphorylation balance in favor of kinase activity, thereby triggering erythrocyte death through the activation of pro-eryptotic kinases. This mechanistic paradigm is elegantly illustrated by three distinct phosphatase inhibitors: NSC-95397, cantharidin, and calyculin A, each targeting different phosphatase families yet converging on common downstream kinase-dependent pathways. All three compounds discussed in this section are experimental compounds included to illustrate the mechanistic principle that phosphatase inhibition can trigger eryptosis.

***NSC-95397***, originally characterized as a CDC25B phosphatase inhibitor, provides a compelling example of how blocking upstream phosphatases can initiate the eryptotic cascade. The exposure of human mature erythrocytes to NSC-95397 for 48 h resulted in significant PS exposure, cell shrinkage, and increased cytosolic Ca^2+^ concentration, accompanied by elevated ROS and ceramide abundance [81]. The effect on PS exposure was partially dependent on extracellular Ca^2+^ and was significantly blunted by the PKC inhibitor staurosporine. Although NSC-95397 does not directly inhibit kinases, its action on CDC25 phosphatases, which normally dephosphorylate and inactivate cyclin-dependent kinases, leads to sustained kinase activation. This demonstrates that eryptosis can be triggered not only by direct kinase inhibition but also by the paradoxical activation of kinases through phosphatase blockade, with PKC emerging as a critical downstream effector [82].

***Cantharidin***, a natural toxin isolated from blister beetles, is a well-established inhibitor of protein phosphatase 1 (PP1) and protein phosphatase 2A (PP2A), with PP2A being 5- to 12-fold more sensitive to the compound [83]. Alzoubi et al. (2015) investigated the eryptotic potential of cantharidin and found that a 48-h treatment significantly increased PS exposure, decreased cell volume, and elevated cytosolic Ca^2+^ concentration in human mature erythrocytes, without affecting ceramide or ROS levels [84]. Critically, the up-regulation of PS exposure was abolished by the broad-spectrum kinase inhibitor staurosporine and partially reduced by the p38 MAPK inhibitor skepinone. Removal of extracellular Ca^2+^ did not significantly blunt the response, indicating that while Ca^2+^ influx occurs, the primary driver of eryptosis is downstream kinase activation resulting from phosphatase inhibition. The mechanistic basis for this effect lies in cantharidin’s binding to the catalytic domain of PP1 and PP2A, where its anhydride ring is hydrolyzed and coordinates with active site metal ions, effectively blocking substrate access and tipping the phosphorylation balance toward kinase-mediated signaling [83]. Wang et al. (2017) further confirmed that cantharidin and its analogue ***norcantharidin*** potently inhibit PP1 and PP2A in vivo, with downstream consequences including apoptosis, DNA damage, and, as demonstrated in erythrocytes, eryptosis [85].

***Calyculin A***, a potent inhibitor of PP1 and PP2A isolated from the marine sponge Discodermia calyx, represents an even more potent phosphatase inhibitor with nanomolar efficacy. Almasry et al. (2016) demonstrated that exposure to calyculin A at concentrations as low as 2.5 nM significantly increased PS exposure, decreased cell volume, and elevated cytosolic Ca^2+^ in human mature erythrocytes [86]. The eryptotic effect was significantly blunted by removal of extracellular Ca^2+^, as well as by inhibitors targeting multiple signaling nodes: staurosporine (PKC), SB203580 (p38 MAPK), D4476 (CK1α), and zVAD (caspases). This broad sensitivity to kinase and caspase inhibitors reveals that calyculin A-induced eryptosis operates through a complex signaling network involving Ca^2+^ entry and the coordinated activation of multiple downstream kinases and caspases. The requirement for both Ca^2+^ and kinase activity distinguishes calyculin A from cantharidin, highlighting the diversity of mechanisms even among phosphatase inhibitors.

Taken together, the studies on phosphatase inhibitors reveal a unifying principle: by removing the brakes on phosphorylation, these compounds unleash kinase activity that ultimately executes the eryptotic program. The three inhibitors discussed, NSC-95397 (CDC25 inhibitor), cantharidin (PP1/PP2A inhibitor), and calyculin A (PP1/PP2A inhibitor), all converge on PKC activation and, in most cases, p38 MAPK activation, with Ca^2+^ influx playing a variable but often contributory role. This stands in contrast to the direct kinase inhibitors discussed in previous sections (such as dasatinib, sunitinib, or thioridazine), which block kinase activity rather than promoting it. The paradoxical outcome, that both kinase inhibitors and phosphatase inhibitors can trigger eryptosis, underscores the delicate balance of phosphorylation in erythrocyte homeostasis. It also expands the conceptual framework for understanding drug-induced anemia: any pharmacological agent that disrupts the equilibrium between kinases and phosphatases, whether by direct inhibition of kinases or by inhibition of their counter-regulatory phosphatases, has the potential to trigger suicidal erythrocyte death. The eryptotic machinery is thus responsive not only to the loss of kinase activity but also to the pathological gain of kinase signaling resulting from phosphatase blockade.

### 2.9. Critical Synthesis and Comparative Analysis of Signaling Pathways

The preceding sections systematically catalog the evidence for eryptosis induction by diverse kinase and phosphatase inhibitors. While this comprehensive approach is necessary to illustrate the breadth of the phenomenon, a critical synthesis of the reported findings reveals several important patterns, conflicts, and limitations that warrant explicit discussion.

First, comparative analysis of the signaling pathways engaged by different inhibitor classes reveals a spectrum of mechanisms rather than a single uniform pathway. Many TKIs (dasatinib, sunitinib, afatinib, gefitinib, ceritinib, pazopanib, sorafenib) and some PI3K/AKT/mTOR inhibitors (temsirolimus) converge on Ca^2+^ influx as a central mediator. In contrast, other inhibitors operate through Ca^2+^-independent pathways (lapatinib, ruxolitinib, boswellic acid, deguelin, cepharanthine). Downstream of Ca^2+^, p38 MAPK activation is a common effector in many cases (sunitinib, ruxolitinib, boswellic acid, sclareol), while PKC activation predominates in others (temsirolimus, juglone, costunolide, manumycin A, camalexin). CK1α activation emerges as a recurring theme across diverse inhibitors (ceritinib, gingerol, arctigenin, eriocitrin, galangin, deguelin, sclareol, β-caryophyllene oxide, α-linolenic acid), suggesting that this kinase represents a critical nodal point in the eryptotic machinery regardless of the upstream trigger. This heterogeneity underscores that the eryptotic potential of kinase inhibitors is not a single uniform phenomenon but rather a complex outcome of target-specific and off-target effects on erythrocyte signaling networks.

Second, several conflicting observations merit discussion. The variable role of Ca^2+^ across different TKIs is particularly noteworthy: lapatinib and ruxolitinib operate independently of Ca^2+^ influx, whereas dasatinib, sunitinib, and afatinib require Ca^2+^ entry. Similarly, among PI3K/AKT/mTOR inhibitors, temsirolimus engages Ca^2+^-dependent pathways, while cepharanthine triggers eryptosis through entirely Ca^2+^-independent mechanisms. These discrepancies may reflect differences in chemical structure, off-target binding profiles, or the specific kinases inhibited. The divergent mechanisms observed even within the same inhibitor class highlight that generalizations about class effects should be made with caution.

Third, methodological limitations across studies complicate direct comparisons. Studies vary considerably in compound concentrations, exposure times (ranging from 24 to 48 h), donor variability, and the specific assays used to assess eryptosis hallmarks. While most studies employ annexin-V binding for PS exposure and forward scatter for cell shrinkage, the assessment of Ca^2+^ influx (Fluo3 or Fluo4 fluorescence), oxidative stress (DCFDA fluorescence), and ceramide abundance is less standardized. Furthermore, the majority of studies rely on pharmacological inhibitors to dissect signaling pathways, which, while informative, are subject to off-target effects and incomplete specificity. Only a minority of studies employ genetic approaches (knockout mice) to confirm pathway involvement. For instance, studies on JAK3-deficient mice (jak3-/-) [47], AMPKα1-deficient mice (ampk-/-) [56], and MSK1/2-deficient mice (msk-/-) [87,88] have provided crucial in vivo validation of the regulatory roles of these kinases in eryptosis.

Fourth, the strength of evidence supporting the proposed signaling pathways varies considerably across compounds. For compounds with multiple independent studies and consistent findings (e.g., dasatinib, sunitinib, sorafenib), the evidence can be considered strong. For compounds with only a single study (e.g., BI2536, NSC-95397, many natural products), the evidence is more limited and requires replication. Furthermore, for compounds where evidence is derived from erythroid progenitors or other cell types rather than mature erythrocytes (e.g., LY294002, Wortmannin, MK-2206), the evidence is indirect and should be interpreted with appropriate caution.

**Figure 2:**
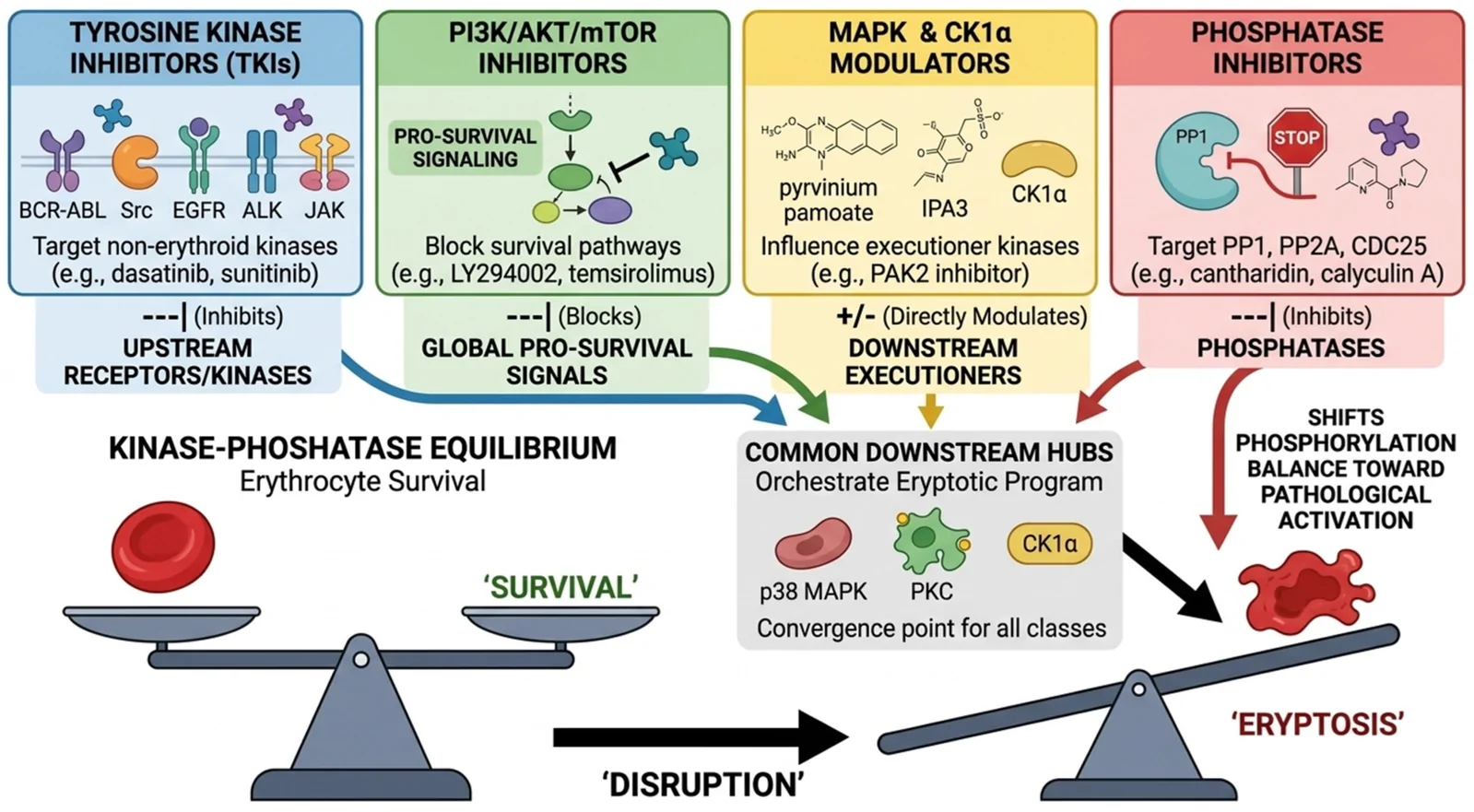
Kinase and phosphatase disequilibrium inducing eryptosis.

Figure 2 and Table 1 summarize the key pharmacological inhibitors discussed in this section, their primary molecular targets, the hallmarks of eryptosis they induce, and the proposed underlying mechanisms.

## 3. Therapeutic Strategies to Mitigate Drug-Induced Eryptosis

**Figure 3:**
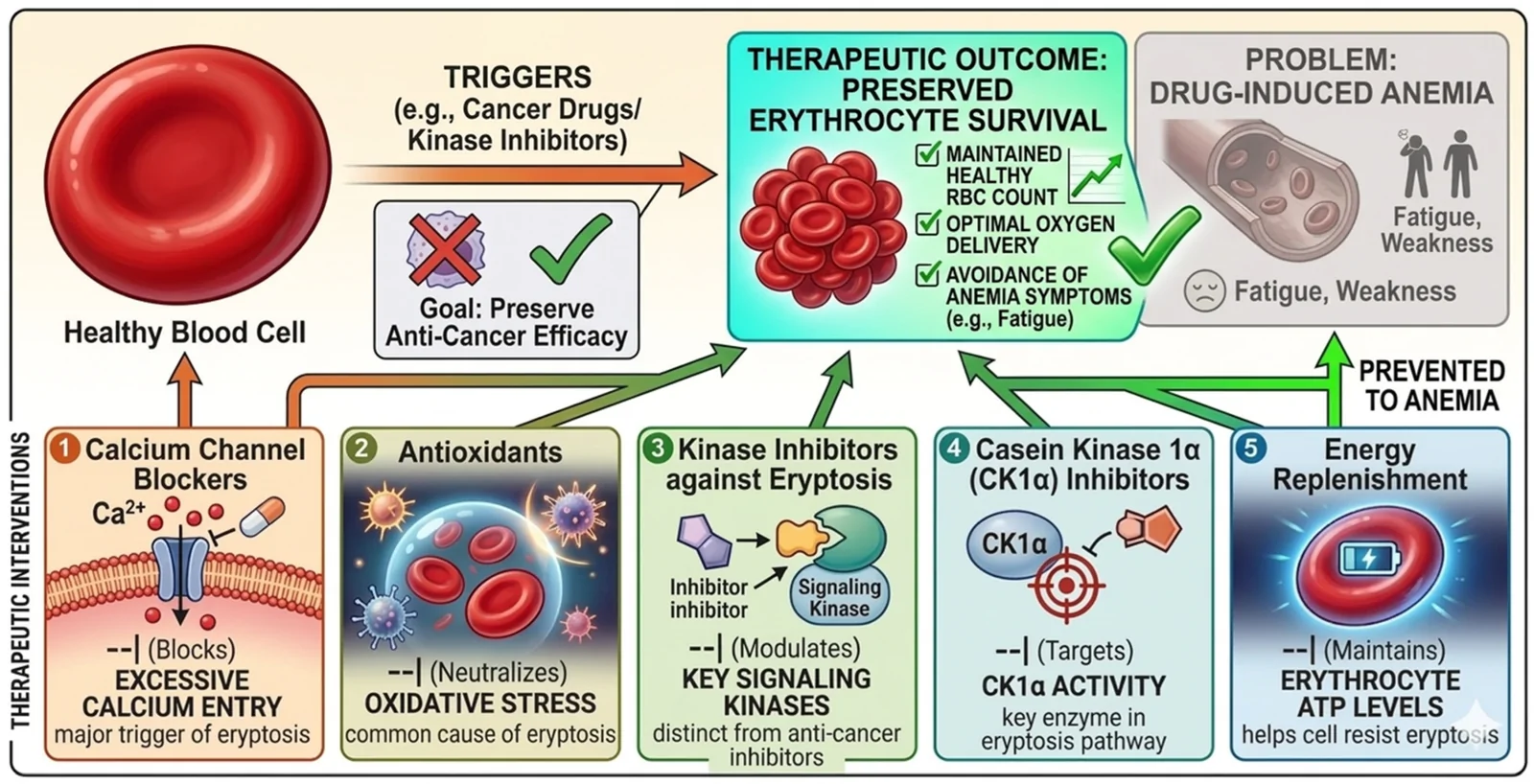
Therapeutic Strategies to Protect Erythrocytes from Drug-Induced Eryptosis.

The recognition that eryptosis contributes to drug-induced anemia opens avenues for therapeutic intervention aimed at preserving erythrocyte survival without compromising the anti-cancer efficacy of kinase inhibitors (Figure 3).

### 3.1. Calcium Channel Blockers

Since Ca^2+^ influx is a central mediator of eryptosis, pharmacological blockade of Ca^2+^-permeable channels represents a rational strategy for preventing drug-induced erythrocyte death. TRPC6 inhibitors, such as lanthanum chloride and SKF-96365, have been shown to blunt eryptosis induced by various stimuli in vitro [6]. However, the clinical utility of such agents is limited by their potential cardiovascular effects and lack of selectivity. The use of clinically approved calcium channel blockers (e.g., verapamil, diltiazem) for this indication remains unexplored and warrants investigation in preclinical models [89].

### 3.2. Antioxidants

Given the prominent role of oxidative stress in many forms of drug-induced eryptosis, antioxidant supplementation has been proposed as a protective strategy. N-acetylcysteine (NAC), a precursor of glutathione, has been shown to significantly reduce PS exposure and cell shrinkage in erythrocytes exposed to sorafenib, dasatinib, and other pro-eryptotic agents [27,90]. Similarly, vitamin C (ascorbate) and vitamin E (α-tocopherol) have demonstrated protective effects in vitro [91,92]. Whether these antioxidants can be safely co-administered with kinase inhibitors without interfering with their anti-tumor activity remains to be determined. Notably, some anticancer therapies rely on oxidative stress to kill cancer cells, and antioxidant supplementation could theoretically reduce treatment efficacy [93].

### 3.3. Kinase Inhibitors That Protect Against Eryptosis

Paradoxically, certain kinase inhibitors have been shown to suppress eryptosis rather than induce it. Volasertib (PLK1 inhibitor) and Reversine (MPS1 inhibitor) blunts eryptosis induced by energy depletion and hyperosmotic shock [94,95]; CDK4 inhibitors (NSC625987, ryuvidine) suppress eryptosis triggered by Cl^−^ removal and oxidative stress [96]; and the CK1α inhibitor D4476 blocks eryptosis induced by glucose depletion [97]. While these agents are not currently used for this purpose, they provide proof-of-concept that pharmacological modulation of the eryptotic machinery is possible. Future drug development efforts could focus on identifying or designing kinase inhibitors with reduced eryptotic liability or incorporating protective moieties into existing agents.

### 3.4. Casein Kinase 1α (CK1α) as a Therapeutic Target

Given the central role of CK1α in mediating eryptosis across diverse stimuli, selective CK1α inhibition represents a promising therapeutic strategy [98]. The CK1α inhibitor D4476 has been shown to protect erythrocytes from eryptosis induced by glucose deprivation, oxidative stress, and several kinase inhibitors [97]. The development of CK1α inhibitors with favorable pharmacokinetic properties and minimal off-target effects could provide a means to mitigate drug-induced eryptosis while preserving the anti-cancer activity of co-administered agents. However, the potential impact of CK1α inhibition on cancer cell survival must be carefully evaluated, as CK1α also plays roles in tumor biology [99].

### 3.5. Energy Replenishment

Several studies have demonstrated that energy depletion is a critical contributor to eryptosis induced by various agents, including cepharanthine, arctigenin and deguelin [54,69,72]. Supplementation with adenine, guanosine, and glucose has been shown to protect erythrocytes from PS exposure and hemolysis in vitro [54,72]. These findings raise the possibility that metabolic support, perhaps through nutritional supplementation, could reduce the eryptotic burden in patients receiving TKI therapy. Clinical trials evaluating the impact of such interventions on anemia outcomes are warranted.

## 4. Future Research Directions

Despite the significant advances summarized in this review, several critical questions remain unanswered and represent important avenues for future research.

### 4.1. Mechanistic Heterogeneity

The diversity of mechanisms by which different kinase inhibitors induce eryptosis, ranging from Ca^2+^-dependent to Ca^2+^-independent, from p38-mediated to PKC-mediated, suggests that no single pathway explains all cases. High-throughput, unbiased approaches such as phosphoproteomics and metabolomics could identify common nodes of convergence across diverse inducers, potentially revealing novel therapeutic targets [56]. Additionally, the identification of genetic determinants of susceptibility to drug-induced eryptosis (e.g., polymorphisms in genes encoding TRPC6, CK1α, or antioxidant enzymes) could enable personalized risk stratification [73].

### 4.2. In Vivo Validation

The majority of studies reviewed here were conducted in vitro using erythrocytes from healthy donors. While these studies provide valuable mechanistic insights, they do not fully recapitulate the complex in vivo environment, including the effects of drug metabolism, protein binding, and interactions with other cell types. Development of animal models that faithfully reproduce human eryptosis and allow for longitudinal assessment of erythrocyte survival is needed to validate the clinical relevance of in vitro findings [87,88,100]. Existing in vivo evidence, including the demonstration of elevated PS-exposing erythrocytes in sorafenib-treated patients [27] and the protection from eryptosis in JAK3-deficient mice [47], supports the translational relevance of these findings, though further studies are needed. Transgenic mice expressing human drug targets and pharmacokinetic/pharmacodynamic modeling could bridge the gap between bench and bedside.

### 4.3. Clinical Correlates

While several studies have documented elevated PS-exposing erythrocytes in patients receiving TKI therapy [25,27,40], prospective studies correlating eryptosis biomarkers with clinical outcomes, including hemoglobin decline, transfusion requirements, quality of life, and thromboembolic events, are lacking. Such studies are essential to establish the clinical utility of eryptosis monitoring and to identify thresholds for intervention [33].

### 4.4. Interventional Trials

Ultimately, the clinical relevance of drug-induced eryptosis will be determined by whether interventions that block eryptosis can reduce the burden of anemia in patients receiving kinase inhibitors. Pilot studies evaluating the safety and efficacy of antioxidants, calcium channel blockers, or CK1α inhibitors as adjunctive therapy in patients with TKI-associated anemia are needed [71]. Such trials must carefully balance the potential benefits of preserving erythrocyte survival against the risk of interfering with the anti-cancer activity of the primary therapy [54].

### 4.5. Impact on Treatment Continuation and Outcomes

Severe anemia is a common cause of dose reduction, treatment interruption, or discontinuation of kinase inhibitor therapy, potentially compromising long-term outcomes [21]. If eryptosis is a major contributor to anemia, then interventions that mitigate eryptosis could enable patients to remain on optimal doses of their anti-cancer therapy, potentially improving survival. This hypothesis represents a compelling rationale for continued investigation of eryptosis in the context of targeted cancer therapy [101].

## 5. Conclusions

The discovery that a wide range of pharmacological kinase and phosphatase inhibitors induce eryptosis has transformed our understanding of drug-induced anemia. Rather than being solely a consequence of bone marrow suppression, anemia in patients receiving these agents may also be contributed to by the accelerated destruction of mature circulating erythrocytes through a highly regulated, programmed cell death pathway. However, the quantitative contribution of eryptosis to clinical anemia, relative to impaired erythropoiesis, remains to be established in prospective patient studies. The clinical implications of this phenomenon are far-reaching, affecting patient management, risk stratification, and the development of novel therapeutic strategies to preserve erythrocyte survival without compromising anti-cancer efficacy.

The diversity of mechanisms by which different agents trigger eryptosis, spanning Ca^2+^-dependent and Ca^2+^-independent pathways, direct kinase inhibition and phosphatase blockade, and the engagement of multiple downstream signaling nodes including p38 MAPK, PKC, and CK1α, underscores the complexity of erythrocyte biology and the vulnerability of these anucleate cells to pharmacological perturbation. At the same time, this diversity reveals multiple potential points of therapeutic intervention, from calcium channel blockade to antioxidant supplementation to targeted inhibition of pro-eryptotic kinases such as CK1α.

As the development of novel kinase inhibitors continues to accelerate, the incorporation of eryptosis screening into preclinical safety assessments, the validation of biomarkers for clinical monitoring, and the conduct of interventional trials to mitigate drug-induced eryptosis represent critical priorities. By integrating mechanistic insights with clinical observation, we can hope to reduce the burden of anemia in patients receiving life-saving targeted therapies, improving both quality of life and treatment outcomes.

## Figures and Tables

**Table 1:** Key kinase and phosphatase inhibitors discussed in this review that trigger eryptosis, their primary molecular targets, the hallmarks of eryptosis they induce, and the proposed underlying mechanisms.

Inhibitor	Primary Target(s)	Eryptosis Hallmarks	Proposed Mechanism	Key Reference
BI2536 (c)	PLK1, PLK2, PLK3	PS exposure, cell shrinkage	Ca^2+^ influx, ceramide accumulation	[39]
Dasatinib (a)	BCR-ABL, Src	PS exposure, cell shrinkage	Ca^2+^ influx, membrane permeabilization, caspase-3 activation	[25]
Sunitinib (a)	VEGFR, PDGFR	PS exposure, cell shrinkage	Ca^2+^ influx, p38 MAPK activation, caspase activation	[40]
Afatinib (a)	EGFR, HER2	PS exposure, cell shrinkage	Ca^2+^ influx, oxidative stress, ceramide	[41]
Gefitinib (a)	EGFR	PS exposure, cell shrinkage	Ca^2+^ influx, oxidative stress	[42]
Lapatinib (a)	EGFR, HER2	PS exposure, cell shrinkage	Ca^2+^-independent	[43]
Ceritinib (a)	ALK	PS exposure, cell shrinkage	Ca^2+^ influx, PKC/p38/CK1α/caspase activation	[44]
Ruxolitinib (a)	JAK1, JAK2	PS exposure, cell shrinkage	p38 MAPK activation (Ca^2+^-independent)	[45]
Pazopanib (a)	VEGFR, PDGFR, c-Kit	PS exposure, cell shrinkage, hemolysis	Ca^2+^ influx, oxidative stress, ceramide	[46]
Sorafenib (a)	Raf, VEGFR, PDGFR	PS exposure, cell shrinkage, hemolysis	Ca^2+^ influx, oxidative stress	[27]
JAK3 (genetic/model)	JAK3	PS exposure	Energy depletion-induced; JAK3-dependent	[47]
LY294002 (c, ‡)	PI3K	PS exposure, apoptosis	Blocks PI3K/Akt survival pathway	[48]
Wortmannin (c, ‡)	PI3K	PS exposure, apoptosis	Inhibits PI3K/Akt pathway	[50]
MK-2206 (c, ‡)	Akt	PS exposure, apoptosis	Blocks Akt phosphorylation	[51]
Temsirolimus (a)	mTOR	PS exposure, cell shrinkage	Ca^2+^ influx, oxidative stress, ceramide, PKC activation	[53]
Cepharanthine (c)	PI3K, AKT, mTOR, AMPK	PS exposure, cell shrinkage, hemolysis, blebbing	Ca^2+^-independent; p38/CK1α/COX/MLKL/PKC/iNOS	[54]
Pyrvinium pamoate (c)	CK1α activator	PS exposure	Enhances Ca^2+^ influx, activates cation channels	[55]
PAK2 inhibitor IPA3 (c)	PAK2	PS exposure	PAK2 inactivation promotes eryptosis	[56]
R406 (b)	SYK	PS exposure (likely)	Inhibits SYK-dependent signaling	[60]
AG490 (c)	JAK2	PS exposure, apoptosis	Inhibits JAK2; activates GSK3β	[61]
Dorsomorphin (c)	AMPK	PS exposure, cell shrinkage	Disrupts energy homeostasis	[63]
Thioridazine (c)	PI3K, AKT, mTOR	PS exposure, cell shrinkage	Ca^2+^ influx, p38 MAPK activation	[64]
Juglone (c)	PI3K, AKT	PS exposure, cell shrinkage	Ceramide, ATP depletion, PKC activation	[65]
Boswellic acid (c)	IKK, PI3K, AKT, mTOR	PS exposure, cell shrinkage	p38 MAPK activation (Ca^2+^-independent)	[66]
Fucoxanthin (c)	PI3K, AKT, mTOR	PS exposure, cell shrinkage, hemolysis	Ca^2+^ influx, lipid peroxidation	[67]
Gingerol (c)	PI3K, AKT, mTOR (inhibits); CK1α (activates)	PS exposure, cell shrinkage, hemolysis	Ca^2+^ influx, CK1α activation	[68]
Arctigenin (c)	Unknown	PS exposure, cell shrinkage, hemolysis	Ca^2+^ influx, CK1α activation, energy depletion	[69]
Eriocitrin (c)	Unknown	PS exposure, cell shrinkage, hemolysis	Ca^2+^ influx, oxidative stress, CK1α, Rac1	[70]
Galangin (c)	Unknown	PS exposure, cell shrinkage, hemolysis	Ca^2+^ influx, PKC/CK1α/COX/p38/Rac1	[71]
Deguelin (c)	Unknown	PS exposure, hemolysis	Ca^2+^-independent, CK1α activation, ATP depletion	[72]
Costunolide (c)	Unknown	PS exposure, cell shrinkage	PKC-α activation, GSH depletion, G6PDH inhibition	[73]
Sclareol (c)	Unknown	PS exposure, hemolysis	p38 MAPK, CK1α activation (Ca^2+^-independent)	[74]
Embelin (c)	Unknown	PS exposure, hemolysis	Ceramide accumulation	[75]
Phloretin (c)	Unknown	PS exposure	Ceramide accumulation	[76]
Manumycin A (c)	Farnesyltransferase	PS exposure, cell shrinkage, hemolysis	Ca^2+^ influx, PKC, p38 MAPK activation	[77]
Camalexin (c)	Src kinase	PS exposure, cell shrinkage	Ca^2+^ influx, PKC, caspase activation	[78]
β-Caryophyllene oxide (c)	Unknown	PS exposure, cell shrinkage, hemolysis	Ca^2+^ influx, oxidative stress, CK1α, PKC, MLKL	[79]
α-Linolenic acid (c)	Unknown	PS exposure, hemolysis	Ca^2+^ influx, oxidative stress, CK1α, MLKL	[80]
NSC-95397 (c)	CDC25 phosphatase	PS exposure, cell shrinkage	Ca^2+^ influx, ROS, ceramide, PKC activation	[81]
Cantharidin (c)	PP1, PP2A	PS exposure, cell shrinkage	Ca^2+^ influx, staurosporine- and p38-sensitive	[84,85]
Calyculin A (c)	PP1, PP2A	PS exposure, cell shrinkage	Ca^2+^ influx, PKC, p38, CK1α, caspase activation	[86]

(a) Clinically approved in at least one major regulatory jurisdiction (FDA, EMA, or equivalent). (b) Investigational/clinical development (approved in some jurisdictions or in clinical trials). (c) Experimental compound used primarily for mechanistic studies. (‡) Indirect evidence: Studies performed in erythroid progenitors, hematopoietic cells, or cancer cell lines, not mature erythrocytes.

## Data Availability

No new data were created or analyzed in this study. Data sharing is not applicable to this article.
